# It is not always Tuberculosis! A case of pulmonary cryptococcosis in an immunocompetent child in Uganda

**DOI:** 10.4314/ahs.v21i3.5

**Published:** 2021-09

**Authors:** Irene Nakatudde, Phillip Kasirye, Sarah Kiguli, Philippa Musoke

**Affiliations:** 1 Department of Paediatrics and Child Health, College of Health Sciences, Makerere University; 2 Mulago National Referral Hospital, Kampala, Uganda; 3 Makerere University-Johns Hopkins University Research Collaboration, Kampala, Uganda

**Keywords:** Pulmonary cryptococcosis, immunocompetent, children, Uganda

## Abstract

Pulmonary cryptococcosis is rare in immunocompetent individuals. Limited data exist regarding its occurrence in children, especially in developing countries. This case report describes an 8-year-old HIV-negative child with pulmonary cryptococcosis, previously diagnosed and treated for tuberculosis twice without improvement. Fine needle aspiration biopsy confirmed the diagnosis of pulmonary cryptococcosis and serum cryptococcal antigen test was positive. The child improved on amphotericin and fluconazole treatment. Despite the limited diagnostic capacity in many resource-constrained settings like Uganda, this case report highlights the need to investigate other causes of pneumonia in immunocompetent children that are not improving on conventional antimicrobial treatments.

## Introduction

Cryptococcosis is an invasive fungal disease caused by an encapsulated yeast *Cryptococcus neoformans*^1^. Cryptococcosis commonly affects immunocompromised individuals with HIV and AIDS, hematological malignancies, diabetes mellitus and other conditions that impair T-cell immunity^1^. It occurs less frequently in immunocompetent persons and is rare in children^1^–^3^. Cryptococcal organisms have a predilection for pulmonary and central nervous systems (CNS)^3^. Immunosuppressed children often present with disseminated infection involving the CNS as opposed to immunocompetent children who may be asymptomatic or show mild symptoms^2^. Pulmonary cryptococcosis (PC) in immunocompetent children is rarely diagnosed and its prevalence in non-HIV-infected children in Uganda is unknown^4^. Reasons for infrequent diagnosis include its rare occurrence, atypical presentation, low index of suspicion among health workers and limited diagnostic capacity especially in resource-limited settings^5^. As such, PC diagnosis is frequently delayed, often resulting in complications. To our knowledge, no case of PC in an immunocompetent child has been documented in our setting. We describe a case of PC in an 8-year-old male HIV-negative child initially treated as tuberculosis with the hope of raising its index of suspicion among clinicians in Uganda and other resource-limited settings.

## Case report

**Figure 1:**
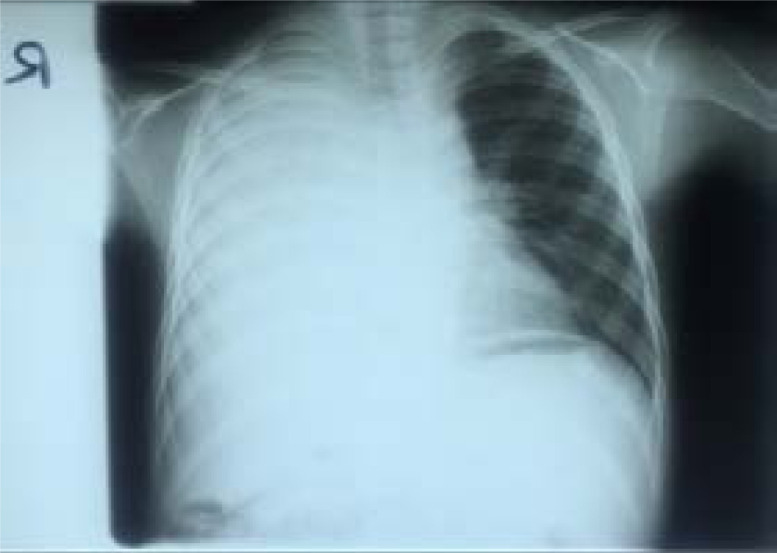
Chest radiograph showing a homogeneous opacity in the right lung

An 8-year-old male presented with productive cough, low-grade fevers, night sweats, weight loss, and on and off right-sided chest pain for one year. He developed difficulty in breathing (DIB) and facial puffiness two weeks prior to admission. There was no history of hemoptysis or trauma and his micturition habits were normal. Review of other systems was unremarkable. History of contact with an adult with chronic cough or tuberculosis (TB) was negative. Previously, he had been managed for TB at a local health facility with minimal improvement. He had no chronic illnesses, drug or food allergies. Although, the mother was a peasant farmer, there was no contact with birds such as pigeons. Physical examination revealed a fully conscious, well-nourished child in mild respiratory distress (respiratory rate 40b/m). He was febrile (Temperature 37.8°C), SpO2 93% room air. He had no cyanosis, lymphadenopathy, finger clubbing or pedal edema. Positive findings on systemic examination were chest asymmetry with a slight bulge on the right side and therapeutic marks at the lower third of the sternum. Chest movement and expansion was reduced on the right side with tracheal deviation to the right, no palpable masses or tenderness. Air entry was reduced on the same side, with a dull percussion note, increased vocal resonance and bronchial breathing; the left side was normal. Other systems were normal. A provisional diagnosis of lobar pneumonia was made and he was started on IV antibiotics with ceftriaxone 2 g once daily for one week. Investigations done included a complete blood count (CBC), which showed leukocytosis (WBC 12.3 × 10^3^/µL) with predominant neutrophils and mild anaemia (Hb 10.7 g/dl). Renal function tests (RFTs), liver function tests (LFTs), urine and stool analysis were normal. GeneXpert MTB/RIF, Mantoux test, HIV antibody test and DNA-PCR were negative, CD4+ T cell count 1,199 cells/L. Serum beta hCG, alpha-fetoprotein and LDH were normal. The chest X ray (CXR) showed a large homogenous opacity on the right side (Figure 1).

**Figure 2:**
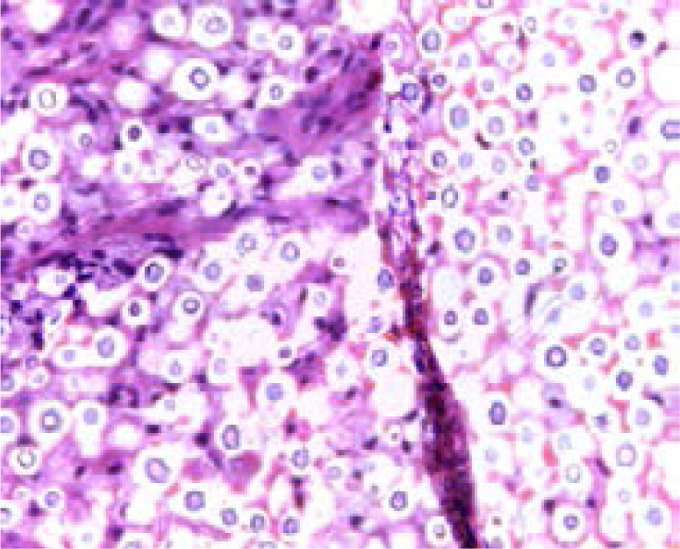
H&E stain of the lung biopsy showing encapsulated yeasts of *Cryptococcus*

Sputum analysis showed gram-negative rods of Klebsiella Pneumonia sensitive to piperacillin-tazobactam, no acid-fast bacilli (AFBs) were seen; therefore, he was initiated on IV piperacillin-tazobactam 1g thrice daily for two weeks. Given that investigations for PTB were negative and no clinical improvement was noted on IV antibiotics, additional investigations were done. These included, blood culture and sensitivity; chest computed tomography (CT) scan and an ultra-sound scan guided lung biopsy. Histological examination of the tissue with Hematoxylin and Eosin (H&E) stain showed encapsulated yeasts of Cryptococcus (Figure 2) with no malignant cells. Additionally, fungal blood cultures revealed creamy-colored colonies that were indicative of Cryptococcus. However, final species identification was not done due to unavailability of reagents.

**Figure 3:**
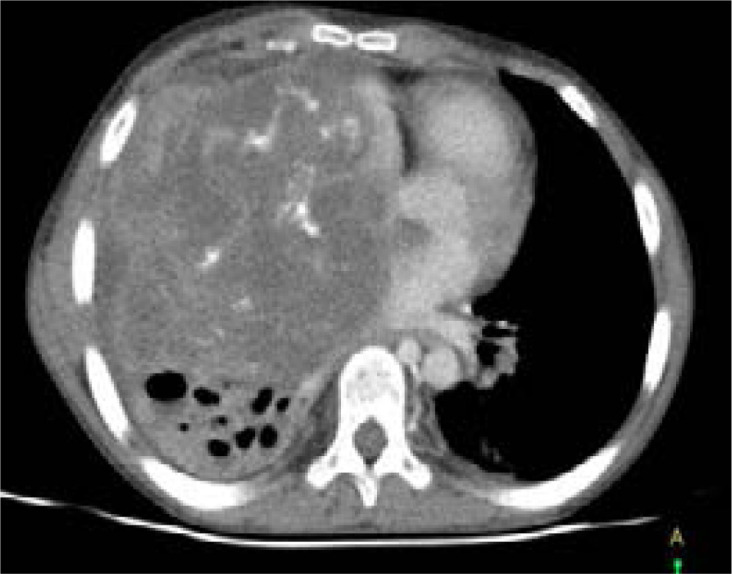
Contrasted axial chest CT image showing an extensive hetero-complex mass in the right hemi-thorax with pericardial invasion

Further investigations were done to determine extent of disease spread. The serum cryptococcal antigen (CrAg) test was positive. The chest CT scan showed an extensive hetero-complex mass in the right hemi-thorax with pericardial invasion. The residual lung tissue was consolidated with multiple cysts (Figure 3). Similarly, the cardiac echo showed a large mass infiltrating the right lung and right atrium without other abnormal findings. The ECG and abdominal ultrasound sound scan were normal. Cerebral spinal fluid (CSF) analysis showed raised protein 1.179g/dl, low glucose 0.1 mmol/L, total WBC 5 cells/uL, and no organisms on Gram stain. However, India ink staining, CSF CrAg and cultre were not done. Given these findings, he was initiated on IV amphotericin B 0.7 mg/kg once daily for 14 days for PC. The cardiothoracic surgeons recommended surgery, however, the child's caretaker declined opting for medical treatment first. He improved and was discharged on oral fluconazole 200 mg once daily. The child did not attend follow-up appointments and was lost to follow-up.

## Discussion

The majority of cryptococcal infections have been reported in immunocompromised adults with a positive HIV status^4^. However, this case report shows that cryptococcosis infection can occur in immunocompetent HIV-negative children. Pulmonary cryptococcosis is commonly acquired by inhalation of the fungal spores contained in the excretion of birds such as pgeons^1^, exposure that was absent in this child. In addition, he did not have the common risk factors for cryptococcal disease, such as diabetes mellitus, cancer, immunotherapy or chronic corticosteroid use that would have predisposed him to the infection. Clinical descriptions of PC in immunocompetent children are quite limited and not well understood, however, it has been noted to commonly occur in children aged 5–17 years and males are more affected, just like in this case^3^, ^6^. Immunocompetent children often have asymptomatic infections that resolve spontaneously. Symptomatic patients like this child frequently present with non-specific symptoms of cough, fever, pleuritic chest pain and constitutional symptoms^1^, ^7^, symptoms that were all present in this child. This clinical presentation does not differ from other differential diagnoses like tuberculosis and malignancies that are more common in our setting than PC. Therefore, it was not surprising that he was initially misdiagnosed as tuberculosis at the local health facility.

At these facilities, given the limited investigative capacity, diagnosis of tuberculosis in children relies most on clinical presentation, a criterion this child met. However, use of clinical symptomatology alone for diagnosis may limit a clinician's ability to investigate a patient extensively to guide early initiation of appropriate treatment for other diseases. Delay in diagnosis may result in fatal consequences as documented in other case reports among adults^8^, ^9^. Diagnostic tools for PC include histology, fungal cultures, serum CrAg and imaging^7^. Radiological features of PC in immunocompetent children are variable. The most common findings are solitary or few well-defined, non-calcified nodules on chest CT scan^7^. This was contrary to what was seen in this child, who had a large calcified consolidated mass in the right hemi-thorax. However, imaging is non-specific and often result in misdiagnosis of PC^10^. Therefore, definitive diagnosis is established by sputum culture or histologic examination of lung tissue^7^. In this case, fine needle aspiration and cytology (FNAC) confirmed the diagnosis, which aided in instituting the appropriate antifungal treatment. Serum CrAg is also an important diagnostic test although it may have low sensitivity in immunocompetent hosts like this child^7^. Despite this, it remains the investigation of choice, as fungal sputum cultures and FNAC are not readily accessible in our setting. Therefore, we recommend serum CrAg screening as part of the diagnostic work up for children with severe pneumonia not improving on antimicrobial therapy irrespective of their immune status. A malignancy was suspected given the history and CXR findings; however, laboratory and histology findings were able to rule this out. Initially, PC was not considered as a differential diagnosis due to the child's negative HIV status, despite the CXR features. This highlights the fact that there may be lack of awareness among clinicians of the broader differential diagnoses in patients with abnormal radiological findings without identifiable risk factors for pulmonary opportunistic infections^11^. Therefore, cryptococcosis should be considered a differential diagnosis in pulmonary lesions on CXR irrespective of a child's immunological status. Tests to confirm CNS involvement were not performed due to lack of the necessary reagents, leaving the possibility of disseminated disease, given the positive serum CrAg despite the absence of CNS symptoms. Further, highlighting some of the diagnostic challenges encountered in resource-limited settings.

The mode of treatment of PC depends on the severity of the disease in an immunocompetent individual^7^. Daily oral fluconazole is indicated for mild to moderate infection without diffuse infiltrates for duration of 6–12 months. For severe disease with diffuse infiltrates as was the case for this child, induction with flucytosine and amphotericin B for at least 14 days, followed by 6–12 months of maintenance fluconazole is recommended^7^, ^12^. Our patient received amphotericin B and fluconazole, as flucytosine is not readily available in our setting. He responded well to medical treatment as evidenced by the clinical improvement. Close follow up is required to monitor response to treatment and adequate counseling should be provided to ensure compliance to treatment. During follow up, clinical, laboratory and radiological monitoring is recommended. Repeat investigations such as chest CT scan, CXR and serum CrAg should be used for treatment monitoring. Specific antifungal therapy usually results in a negative serum CrAg within one year, however in some individuals it may remain persistently positive up to 2 years^13^, ^14^. Radiological resolution varies, but complete resolution is expected within 1–2 years. However, many patients may be left with pulmonary residual abnormalities for much longer periods of time^13^. Despite counseling, the child was lost to follow-up; therefore, the final outcome was not available.

## Conclusion

Pulmonary cryptococcosis in HIV-negative children in Uganda is either rare or under diagnosed due to low index of suspicion by health workers. Despite the limited diagnostic capacity in our setting, PC should be considered in all children presenting with severe pneumonia not responding to conventional antibiotics irrespective of their immune status. The serum CrAg test is a potential screening tool to enable earlier diagnosis and treatment, preventing morbidity and mortality associated with the disease.
